# Investigating the Roles and Responsibilities of Institutional Signing Officials After Data Sharing Policy Reform for Federally Funded Research in the United States: National Survey

**DOI:** 10.2196/49822

**Published:** 2024-03-20

**Authors:** Jinyoung Baek, Jonathan Lawson, Vasiliki Rahimzadeh

**Affiliations:** 1 Broad Institute Cambridge, MA United States; 2 Center for Medical Ethics and Health Policy Baylor College of Medicine Houston, TX United States

**Keywords:** biomedical research, survey, surveys, data sharing, data management, secondary use, National Institutes of Health, signing official, information sharing, exchange, access, data science, accessibility, policy, policies

## Abstract

**Background:**

New federal policies along with rapid growth in data generation, storage, and analysis tools are together driving scientific data sharing in the United States. At the same, triangulating human research data from diverse sources can also create situations where data are used for future research in ways that individuals and communities may consider objectionable. Institutional gatekeepers, namely, signing officials (SOs), are therefore at the helm of compliant management and sharing of human data for research. Of those with data governance responsibilities, SOs most often serve as signatories for investigators who deposit, access, and share research data between institutions. Although SOs play important leadership roles in compliant data sharing, we know surprisingly little about their scope of work, roles, and oversight responsibilities.

**Objective:**

The purpose of this study was to describe existing institutional policies and practices of US SOs who manage human genomic data access, as well as how these may change in the wake of new Data Management and Sharing requirements for National Institutes of Health–funded research in the United States.

**Methods:**

We administered an anonymous survey to institutional SOs recruited from biomedical research institutions across the United States. Survey items probed where data generated from extramurally funded research are deposited, how researchers outside the institution access these data, and what happens to these data after extramural funding ends.

**Results:**

In total, 56 institutional SOs participated in the survey. We found that SOs frequently approve duplicate data deposits and impose stricter access controls when data use limitations are unclear or unspecified. In addition, 21% (n=12) of SOs knew where data from federally funded projects are deposited after project funding sunsets. As a consequence, most investigators deposit their scientific data into “a National Institutes of Health–funded repository” to meet the Data Management and Sharing requirements but also within the “institution’s own repository” or a third-party repository.

**Conclusions:**

Our findings inform 5 policy recommendations and best practices for US SOs to improve coordination and develop comprehensive and consistent data governance policies that balance the need for scientific progress with effective human data protections.

## Introduction

The rapid growth in human research data, storage, and analysis tools and skilled researchers combined with the declining costs and new federal policies are creating an immense drive for research data sharing [1]. At the same, triangulating human research data of diverse types can accentuate the risks of reidentification when used for secondary research purposes. It also invites the potential for group and other individual dignitary harms that institutional ethics review committees may be underequipped to properly protect participants against [2] or prevented from considering outright based on statutory interpretation of the US Common Rule [3,4].

The lack of standardized methods for accurately tracking the provenance of data and their permitted uses further complicates the problem [5-7]. Most institutional oversight bodies (eg, institutional review boards [IRBs], privacy boards, and data access committees [DACs]) are also ill-equipped to audit whether and how well secondary data uses align with the terms outlined in participants’ initial consent. These inabilities hinder opportunities for transparency, recourse, or accountability if uses are maligned, and gaps in policy as well as tool development leave the research community vulnerable to unintentional data misuse.

The responsibility for overseeing the ethical conduct of research involving human subjects traditionally lies with IRBs. However, the sensitivity and complex nature of data governance in genomics research present a new challenge that can exceed the review capacities and expertise of traditional IRB oversight [2,8-10]. While DACs have emerged as potential solutions to this issue [11], they are not yet widely implemented in many research institutions and are still in the early stages of growth and practice [12].

Despite these challenges, the expectation to share data has broadened [13] under new federal policies (eg, National Institutes of Health [NIH] Data Management and Sharing [DMS] [14]) and is aided considerably by cloud storage and analysis platforms [15]. The new NIH DMS policy came into effect in January 2023. It requires, among other things, that scientific data stemming from NIH-supported projects must be made publicly accessible without delay and with few exceptions. The final DMS policy defines scientific data as “The recorded factual material commonly accepted in the scientific community as of sufficient quality to validate and replicate research findings, regardless of whether the data are used to support scholarly publications. Scientific data do not include laboratory notebooks, preliminary analyses, completed case report forms, drafts of scientific papers, plans for future research, peer reviews, communications with colleagues, or physical objects, such as laboratory specimens” [14]. Emerging cloud computing environments afford new opportunities for tracking, auditing, and enforcing granular permissions [15] for scientific data in ways that were largely impracticable just several years ago. Yet, the broad use of provenance tracking tools continues to lag, creating situations where data stewards impose stricter barriers for access to data once up front, only to loosely permit the secondary use of these same data later in the research data lifecycle [16]. This situation is analogous to a house with a vaulted front door but a wide-open back door.

A clear example is the rigorous process of both a researcher and signing official (SO) agreeing to terms and conditions for the initial secondary access request. Subsequent sharing and use of consented data that transgress the consent, however, would likely go unmonitored by both the DAC approving access and the data recipient’s institution. This reality perversely incentivizes researchers to engage in informal data sharing to sidestep institutional data access review, which risks participants’ rights to privacy and the reputation of the research community. It is our view that limiting informal sharing pathways is consequential for both participant data protection and for maintaining trustworthy data governance practices. Investing resources into optimizing compliant pathways that are easier and more expedient for data requestors without sacrificing participant terms of use should therefore be a high institutional priority.

Research institutions and repository managers could also confront increased liability burdens from inaccurate, inconsistent, or unspecified data use permissions and restrictions [17]. Misattributing use restrictions can result in regulatory noncompliance and instill a lack of confidence among prospective and current research participants that institutions will respect the terms of their data contributions. SOs, who often serve as signatories on data submission and access request agreements, bear significant responsibility for ensuring the appropriate transfer and use of research data within their institutions in this regard [17].

**Figure 1:**
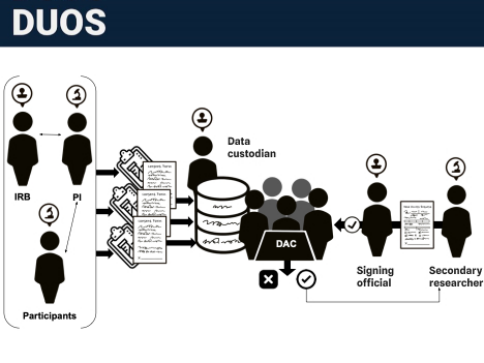
Signing officials act as institutional arbiters of data access between oversight bodies such as DACs and researchers requesting access to research data. DAC: data access committee; DUOS: Data Use Oversight System; IRB: institutional review board; PI: principal investigator.

According to the NIH, SOs have the authority to “legally bind the institution in grant-administration matters by providing signature approval on grant application submissions” [18]. A graphical diagram of where SOs intervene on the data sharing and management processes is depicted in Figure 1. SOs further monitor “grant-related activities within the extramural organization” to ensure compliance with all grant requirements. Although SOs play a leadership role in research compliance, we know very little about the ways in which new data sharing requirements for researchers affect their scope of work and data governance responsibilities in the wake of new agency policies.

This descriptive survey addresses a significant knowledge gap about the roles of US SOs, their knowledge about where research data from extramurally funded projects are deposited, how these data are accessed, and where data can be found after grant funding sunsets. Better understanding the roles and capabilities of SOs is important for identifying where institutional gatekeepers can intervene along the research data lifecycle to improve data provenance tracking and ethical data reuse in light of new NIH data sharing requirements [14]. We explore issues surrounding data sensitivity, consent tracking, institutional responsibilities, and liability considerations of SOs. As such, we seek to underscore the urgency of improving the distribution and coordination of institutional oversight bodies responsible for research data governance involving humans.

## Methods

### Study Design

We designed an anonymous survey of institutional SOs (Multimedia Appendix 1). The purpose of this survey study was to establish a baseline of existing institutional policies and practices as well as how these may change in light of new data sharing and management requirements for federally funded research in the United States. Survey items probed where data generated from extramurally funded research are deposited, how researchers outside the institution access these data, and what happens to these data after extramural funding ends.

### Recruitment

Survey respondents were recruited from the websites of numerous institutions in the United States that receive funding from the NIH. We identified active SOs and used publicly available email addresses to contact them. Links to participate in the web-based survey were sent via email to each identified contact, and we used the software platform Qualtrics to administer the survey.

### Ethical Considerations

This study was exempted by the Broad Institute of MIT and Harvard Office of Research Subject Protection. All survey responses were anonymized. Before advancing to the survey itself, respondents were presented with a detailed description of the study, its purpose, realistic risks and benefits of completing the survey, as well as information regarding the investigators and the funders before advancing to the survey itself. Consent to participate was indicated if respondents proceeded to complete the survey after reviewing the above study information. Respondents were not compensated for their participation.

## Results

The survey took approximately 5 minutes to complete and probed SOs about the impacts of the new DMS policy on their institutional tasks and responsibilities. The survey response rate was 19%: a total of 56 participants (of 287 prospective participants who were contacted) completed the survey between February 6 and April 12, 2023.

The 56 respondents represented institutions including academic-affiliated research institutes (n=43, 77%), academic institutions (n=10, 18%), nonprofit research institutes (n=2, 4%), and a health care or hospital system (n=1, 2%; Table 1).

Of the 56 respondents, 52% (n=29) indicated that they will submit more than 250 grants in the next calendar year (Table 2).

**Table 1:** Institutional types represented by survey respondents.

Institution type	Values (n=56), n (%)
Academic-affiliated research institute	43 (77)
Academic institution	10 (18)
Nonprofit research institute	2 (4)
Health care hospital system	1 (2)

**Table 2:** Estimation of how many NIH^a^ grants will be impacted by new DMS^b^ policy.

Question and response			Values (n=56), n (%)
**How many data-generating NIH grants submitted this year would you estimate will be impacted by the DMS policy at your institution?**			
	1-19	6 (11)	
	20-99	7 (13)	
	100-249	8 (14)	
	Over 250	29 (52)	
	I do not know	6 (11)	

^a^NIH: National Institutes of Health.

^b^DMS: Data Management and Sharing.

Two-thirds (37/56, 66%) of respondents reported that less than 50% of scientific data generated by NIH funding will be controlled access data. When asked about the percentage of controlled access data generated by NIH funding, 46% (n=26) answered “less than 25%,” 20% (n=11) answered “25% to 49%,” 11% (n=6) answered “50% to 74%,” and 5% (n=3) answered “75% to 100%.”

However, there is the potential for additional data sets to be categorized as controlled access data. We found that respondents defaulted to controlled access for data sets when a research participant’s preferences for “open access” versus “controlled access” were unclear in the original consent forms. In total, 27% (13/48) responded that they “always” default to controlled access sharing when the terms of access were unknown, while 52% (25/48) responded they “sometimes” do (Table 3).

**Table 3:** Nearly 80% (48/56) of survey respondents reported they default to controlled access for data where participant terms of use are unknown or ambiguous.

Question and response		Values (n=48), n (%)
**When research participants’ preferences for open access versus controlled access are unclear in the consent, does your institution default to sharing under controlled access guidelines?**		
	Always	13 (27)
	Sometimes	25 (52)
	Rarely	9 (19)
	Never	1 (2)

The respondents reported that most investigators will not only deposit data into “an NIH-funded repository (eg, dbGaP, AnVIL, and BioDataCatalyst)” to meet the DMS requirements but also within the “institution’s own repository” as well as “a third-party repository (eg, Generalist Repository Ecosystem Initiative repositories, Terra, and Data Use Oversight System).” For respondents who reported depositing in an institutional repository, a combined 62% (22/35) reported there was no process for researchers external to the institution to view, request, or access data or that they did not know whether such a process existed (Table 4). Moreover, 77% (10/13) responded that they were unaware if data needed to be downloaded for analysis as opposed to in an analytic sandbox with a security perimeter, in the cloud, or some other secured analytics platform.

**Table 4:** Survey respondents reported whether they knew of a clear process for how external parties request access to data managed by the institution.

Question and response		Values (n=35), n (%)
**Is there a clear process for researchers from other institutions to view, request, and access data (if approved)?**		
	Yes	13 (37)
	No	7 (20)
	I do not know	15 (43)

For respondents who reported data deposits into a third-party repository, nearly 69% (20/29) said that their institution will keep a copy of the data (Table 5). In total, 31% (9/29) of the respondents who selected “a third-party repository” as a chosen platform (Table 6) reported that they are unsure about what will happen to the data at the end of the funded storage period paid for by the DMS allocated costs in each grant. The results suggest that there is a lack of clarity on the management and sharing of the data after the funded period for storage ends. This lack of clarity could mean a significant divergence in the life span and accessibility of publicly funded research data beyond the limited funded periods of storage made possible by DMS funding.

**Table 5:** Signing officials reported that their institution keeps a copy of research data in addition to other data submissions to external repositories.

Question and response			Values (n=29), n (%)
**Will your institution keep a copy of the data?**			
	Yes	20 (69)	
	No	2 (7)	
	I do not know	7 (24)	

**Table 6:** US signing official perspectives on the types of repositories into which investigators deposit research data to comply with the new DMS^a^ policy.

Question and response			Values (n=115), n (%)
**What types of repositories will investigators in your institution deposit into to meet DMS policy requirements?**			
	Your own institution’s data repository	36 (31)	
	An NIH^b^ repository (eg, dbGaP, AnVIL, and BioDataCatalyst)	48 (42)	
	A third-party repository (eg, GREI^c^ repositories, Terra, and DUOS^d^)	29 (25)	
	Other	2 (2)	

^a^DMS: Data Management and Sharing.

^b^NIH: National Institutes of Health.

^c^GREI: Generalist Repository Ecosystem Initiative.

^d^DUOS: Data Use Oversight System.

Respondents felt that principal investigators (PIs) were best positioned to determine where data should be stored to comply with the DMS policy (Table 7). Nearly 58% (38/66) reported that “individual investigators” should decide on which repositories to submit data, while other parties included “chief compliance officer,” “SOs,” “chief information or technology officer,” and “librarians.” Few mentioned that the decision-making should be a shared responsibility among various stakeholders including PIs, IT officers, and SOs.

**Table 7:** Signing officials predominately felt that the project principal investigator was best positioned to decide where research data should be deposited.

Question and response		Values (n=66), n (%)
**Who will decide where data are deposited to meet** **Data Management and Sharing** **policy requirements?**		
	Individual investigators	38 (58)
	Chief compliance officer	5 (8)
	Signing officials	5 (8)
	Chief information or technology officer	7 (11)
	Librarians	6 (9)
	Shared responsibilities among various stakeholders	2 (3)
	I do not know	2 (5)

When asked about who will oversee DMS policy compliance, 33% (19/56) reported that “SOs” should primarily serve that role, while 17% (10/56) mentioned “chief information or technology officer,” 16% (9/56) said “chief compliance officer,” and 9% (5/56) said “PIs.” Approximately 10% (6/56) noted that DMS policy compliance is responsible for multiple institutional offices.

Only 23% (12/52) reported that their institutions track where data from federally funded projects would be deposited, and nearly 37% (19/52) reported they are unaware whether such a tracking system exists. For respondents who reported a lack of a tracking system, 29% (n=6) reported their institutions plan to set up such a system. These data suggest nearly 77% (n=15) of institutions do not have a searchable method for determining where scientific data have been stored to track compliance with the new DMS policies (Table 8).

**Table 8:** Results from 52 survey respondents indicating that more than 77% (n=15) do not track where research data are deposited or are unaware of such tracking at their institution.

Question and response		Values (n=52), n (%)
**Does your institution have a way of tracking into which repositories investigators have deposited data to meet** **Data Management and Sharing** **policy requirements?**		
	Yes	12 (23)
	No	21 (40)
	I do not know	19 (37)

## Discussion

### Principal Findings

Our survey data suggest that SOs approve data deposit into various repositories to comply with the DMS policy but may be unclear about how data stored within the institution are accessed or analyzed by researchers outside the institution. SOs report that many investigators will deposit data into both an NIH-funded repository to meet the DMS requirements and in their institution’s own repository, as well as sometimes in a third-party repository. Such duplicitous deposit has consequences for data storage, security, and costs, which are underacknowledged in the literature.

Investigators may store data in an institutionally controlled repository to comply with new DMS requirements and institutional data retention policies. However, these data may be more difficult for external investigators working in similar fields to find, access, and share in practice and thus could conflict with the FAIR (findability, accessibility, interoperability, and reusability) principles [19]. Storing the same data set in more than 1 repository or database effectively doubles the storage space and costs without the added benefit of improved ease or efficiency of access insofar as institutions rely on commercial companies to provide storage services. While storage costs are typically reimbursed only once as a dedicated line item in grant budgets, commercial service providers profit twice: once for services rendered to store data in NIH-controlled repositories and also for storing these same data on institutional servers or in institutionally controlled databases.

This double storage problem also leads to inconsistent standards for data access management. Access requests for data hosted within NIH repositories and knowledge bases undergo more standardized processing by dedicated staff who review applications and verify authorized users. Data hosted on local institutional servers, in contrast, tend to be more liberally distributed to nonauthenticated users and governed post facto, if at all.

Few SOs know what happens to research data at the end of the contract or storage period for data stemming from federally funded research under the new DMS budget allocations. The most frequently cited reason was that legal or contractual obligations for different data types preclude any one uniform procedure for data handling. Sunsetting projects also threaten the availability of data in perpetuity when allowed costs for data management end. The lack of familiarity or knowledge of access processes outside the institution impedes SO’s abilities to promote sustained use of the data resource longer term.

Less than a quarter (12/25, 23%) of SOs work at institutions that track where data from federally funded projects have been deposited to meet DMS policy requirements. Tracking where data are deposited is critical to verifying compliance with the DMS policy as well as ensuring the repository meets participant-defined terms of use. Without knowing where data end up, SOs are unable to communicate to funders about the state of data availability or provide an accurate accounting of institutional resources needed to steward that data.

As a result, SOs defaulted to imposing greater access controls for data where consent was unclear or unspecified. In total, 27% (13/48) of SOs always defaulted to controlled access for data sets where participant consents were unclear or unspecified, which can translate to downstream issues for authorized data access. A precautionary default approach is advantageous for sensitive data sets where access controls are justified and should have been applied in the first place. Applying controlled access defaults for data that should otherwise be open, however, places undue access barriers that hinder compliant research.

SOs are prone to overcontrolling data when there is ambiguity in consent. Data from this survey suggest that more data will undergo controlled access even if permissioned broadly for general research use. Institutional data stewards, including SOs and DACs, will therefore need to manage increased demands for data. Our prior work with DACs showed they see value in testing automated decision support tools and software to improve the efficiency of access requests without sacrificing consistency or quality [16]. SOs participating in this survey were unaware of such tools, creating clear opportunities for targeted outreach and training**.**

More than half (38/66, 58%) of all SOs surveyed considered that PIs were in the best position to make decisions about where data should be deposited. However, prior studies indicate that relying on PIs alone to make such decisions may be unrealistic considering they often call on institutional resources for support [20,21]. IRBs, program officers, and other institutional data stewards could be consulted in the process if membership were expanded to include individuals with data security, management, and privacy expertise [22]. Additional resources should therefore be made available to support deposit decisions, given that research data constitute primary returns on investment for publicly funded research.

The results of our survey should be considered in light of several limitations. Our results corroborate anecdotal evidence we have gathered through our direct engagement with institutional data stewards, but the low survey response limits the generalizability of our conclusions beyond academic research institutions in the United States. We also administered the survey 1 month after the new DMS policy entered into force and before NIH investigators would have received notices of funding for projects that would need to comply with the new DMS requirements. It is possible that the SOs we surveyed may not have had enough time to fully observe the impacts of the new policy on institutional practice and underestimated them as a result.

### Recommendations

We have conducted empirical survey research with 3 key institutional gatekeepers of research data: IRBs, DACs, and now SOs. Our collective findings led us to make 4 recommendations at the institutional level to better support compliant data access and ethical review of data reuse moving forward (Textbox 1).

**Recommendations for institutional signing officials to promote data provenance tracking, data stewardship, and improved observance of secondary data use permissions.**

- Investigators should be encouraged to store data in a centralized location that can be accessible to authorized users at a distance.
- Recognize privileged access rights for original submitters of data for secondary use, provided they comply with data use terms and, where applicable, are approved by an institutional review board.
- Allow principal investigators to negotiate with National Institutes of Health program officers and committees about how research stemming from the use of private data sets should be publicly disseminated.
- Retain a master list of where all research data stemming from funded projects have been deposited.

To prevent paying double for data storage and management costs, investigators should be encouraged to store data in a centralized location that can be accessible to authorized users at a distance. Public-private partnerships with large commercial cloud service providers, including Amazon Web Services and Microsoft Azure, accentuate the immediacy of this problem, considering they are poised to host some of the nation’s largest research data sets in the coming years [23]. This practice would be highly beneficial for cloud-based repositories, for researchers whose preferred repository to deposit data is cloud-based, and for institutions that support researchers who rely on data sets managed in the cloud to secure future research grants. It is said that centralized storage could reduce the risk of data loss if the original files are corrupted. Data storage is also a growing environmental concern, favoring options that lessen the carbon footprint [24].

Facilitating data access in perpetuity is a reasonable benefit to offer researchers who contributed these data in the first place and could, in turn, incentivize more sharing. However, recognizing this privilege also assumes they will always use the data in ethical ways. While researchers could preserve a private copy of the data, a policy that recognizes special rights of access to original submitters of research data could still be considered insofar, as the proposed research uses accords with consent permissions and, where applicable, has been approved by an IRB.

There are many compelling reasons why researchers prefer to use private data sets [25]. Companies protect their proprietary interests in the data sets they generate by not sharing them freely with the research community. NIH-funded researchers who nevertheless opt to use private data sets in their work do so with full knowledge of company policies against broad data sharing but should not be absolved of their responsibilities to comply with the DMS policy. The NIH could consider enforcement alternatives that help to close this loophole, possibly suspending funds for investigators who are noncompliant or negotiating the sharing of summary-level data to the extent feasible and possible. The government should be reimbursed for data management and storage costs in cases where the benefits of research knowledge using private data sets cannot be recovered. Research funding agencies, as well as individual institutions, should have a master list of where all data stemming from funded projects have been deposited. Researchers should easily be able to see where data generated from funded projects have been deposited, with accessible links to those repositories to directly request access. For example, a widget on the existing NIH Research Portfolio Online Reporting Tool could facilitate this.

### Conclusions

The new NIH DMS policy is a significant step toward responsible data sharing, which will lead to accelerated scientific discoveries and innovation. However, it leaves the details of data sharing and management practices to be determined by individual investigators and their respective institutions. This could create inconsistencies and inefficiencies in data governance, as witnessed in the genomic data sharing ecosystem.

Our survey of institutional SOs in the United States demonstrates a lack of clarity in terms of the location and duration of data storage, costs, and data security. Respondents suggested that scientific data generated by NIH funding are stored at multiple locations, including their institutions’ own data repositories, and are often unsure how external researchers gain access. Additionally, our survey shows that there is a pressing need for the development of proper tracking mechanisms to maintain and ensure the integrity of data sets, as many institutions currently do not keep track of where scientific data are stored once funded projects sunset. While more than half of the respondents suggested that PIs would be the sole decision makers on where to deposit data, respondents also acknowledged that compliance with the DMS policy will require institutional support and facilitate coordination between multiple offices.

## Appendix

Multimedia Appendix 1 Anonymous signing official survey.

## Abbreviations

DAC: data access committee
DMS: Data Management and Sharing
FAIR: findability, accessibility, interoperability, and reusability
IRB: institutional review board
NIH: National Institutes of Health
PI: principal investigator
SO: signing official

## Data Availability

The data sets generated and analyzed during this study are available from the corresponding author on reasonable request.
