# Supplementary material for: Investigating the Roles and Responsibilities of Institutional Signing Officials After Data Sharing Policy Reform for Federally Funded Research in the United States: National Survey
Source: JMIR Form Res. 2024 Mar 20;8:e49822. doi: 10.2196/49822 (PMC10993121; doi:10.2196/49822)
Supplement: Supplementary file 1 [file formative_v8i1e49822_app1.docx]

Signing Official Survey

Survey Flow

Block: Default Question Block (1 Question)

Standard: Block 1 (17 Questions)

| Page Break |  |
| --- | --- |

Start of Block: Default Question Block

Q1  

	
		
			 **Research title**
			 Investigating Institutional Perspectives on Data Access & Sharing
		
		
			 **Study team**
			 Jonathan Lawson, Senior Software Product Manager, Broad Institute of MIT and Harvard

			 Jinyoung Baek, MA, Research Associate, Broad Institute of MIT and Harvard
		
		
			 **Study team contact**
			 Jonathan Lawson / jlawson@broadinstitute.org

			 Jinyoung Baek / jbaek@broadinstitute.org
		
	

 

**Description.** The National Institutes of Health (NIH) recently issued the 2023 NIH Policy for Data Management and Sharing (DMS Policy). Under the new policy, all NIH grant applications for projects that generate scientific data are required to submit a Data Management and Sharing Plan, outlining how scientific data and any accompanying metadata will be managed and shared. The DMS Policy will go into effect on January 25, 2023, and we plan to investigate: (i) how researchers and institutions have prepared for their DMS Plans; and (ii) to evaluate the impacts of the NIH DMS policy on institutions’ data management policies and sharing practices.

**Risks and Benefits.** Your participation in the survey is completely voluntary. No identifiable information about you, your institution, or your responses, will be shared outside of the study team. 

If you have questions about this study, please contact the PIs of this study at jlawson@broadinstitute.org or jbaek@braodinstitute.org. If you have questions as a research participant, please contact Broad’s Office of Research Subject Protections at orsp@broadinstitute.org.

**Time Involvement.** This survey will take approximately 5 minutes to complete. 

**Reimbursements.**  If you participate in the study, you will receive a $20.00 gift card which may be redeemed by completing a separate form at the end of the survey. You will not be reimbursed for any additional research-related expenses.

___________________

**By clicking the next button below, you consent to participate in the survey. Please print a copy of this form for your records.** 

End of Block: Default Question Block

Start of Block: Block 1

Q2 **What is your institution type?**

- Government research agency (e.g., National Institutes of Health)  (1) 

- Academic affiliated research institute (e.g., the Broad Institute of MIT and Harvard)  (2) 

- Non-profit research institute (i.e., affiliated with neither government nor academic institution)  (3) 

- Corporate, commercial, or other for-profit institution (e.g., Pfizer)  (4) 

- Healthcare/hospital system  (5) 

- Consortium (e.g., Psychiatric Genomics Consortium)  (6) 

- Other (e.g., single study/research group)  (7) __________________________________________________

| Page Break |  |
| --- | --- |

Q3 
**The NIH DMS Policy**

 

The National Institutes of Health (NIH) issued the 2023 NIH Policy for Data Management and Sharing (DMS Policy) to promote the management and sharing of scientific data generated from NIH-funded or conducted research, taking effect on January 25, 2023. This Policy establishes the requirements of submission of Data Management and Sharing Plans and compliance with NIH Institute, Center, or Office (ICO)-approved Plans. It also emphasizes the importance of good data management practices and establishes the expectation for maximizing the appropriate sharing of scientific data generated from NIH-funded or conducted research, with justified limitations or exceptions. This Policy applies to research funded or conducted by NIH that results in the generation of scientific data.

| Page Break |  |
| --- | --- |

Q4 **This year, how many data-generating NIH grants submitted would you estimate will be impacted by the DMS Policy at your institution?**

- 1 to 19  (1) 

- 20 to 99  (2) 

- 100 to 249  (3) 

- Over 250  (4) 

- I don't know  (5) 

Q5 **What percentage of scientific data generated by NIH funding at your institution, would you estimate to be controlled-access data (not open-access)?**

- Less than 25%  (1) 

- 25 to 49%  (2) 

- 50 to 74%  (3) 

- 75 to 100%  (4) 

- I don't know  (5) 

| Page Break |  |
| --- | --- |

Q6 **What types of repositories will investigators in your institution deposit into to meet DMS Policy requirements?**

- Your own institution’s data repository  (1) 

- An NIH repository (e.g., dbGaP, AnVIL, BioDataCatalyst)  (2) 

- A third party repository (e.g., GREI repositories, Terra & DUOS)  (3) 

- Other  (4) __________________________________________________

Display This Question:

If Q6 = Your own institution’s data repository

Q7 **Is there a clear process for researchers from other institutions to view, request, and access data (if approved)?**

- Yes  (1) 

- No  (2) 

- I don't know  (3) 

Display This Question:

If Q7 = Yes

Q8 **Does your institution's repository require the data to be downloaded for analysis?**

- Yes  (1) 

- No  (2) 

- I don't know  (3) 

Display This Question:

If Q6 = A third party repository (e.g., GREI repositories, Terra & DUOS)

Q9 **Will your institution keep a copy of the data?**

- Yes  (1) 

- No  (2) 

- I don't know  (3) 

Display This Question:

If Q9 = No

Or Q9 = I don't know

Q10 **What will happen to the data at the end of the contract/funded storage period paid for by the DMS allocated costs in each grant?**

- The data will be deleted  (1) 

- The repository will take ownership of the data  (2) 

- The data will be returned to our institution  (3) 

- I don't know  (4) 

- Other  (5) __________________________________________________

Q11 **Does your institution have a way of tracking into which repositories investigators have deposited data to meet DMS Policy requirements?**

- Yes  (1) 

- No  (2) 

- I don't know  (3) 

Display This Question:

If Q11 = No

Q12 **Does your institution envision setting up a way to track dataset submissions to meet DMS Policy requirements?**

- Yes  (1) 

- No  (2) 

- I don't know  (3) 

| Page Break |  |
| --- | --- |

Q13 **When research participants’ preferences for open-access vs. controlled-access is unclear in the consent, does your institution default to sharing under controlled-access guidelines?**

- Always  (1) 

- Sometimes  (2) 

- Rarely  (3) 

- Never  (4) 

Q14 **Are you familiar with any of the following tools for data storage and sharing?**

- NHGRI AnVIL  (1) 

- Terra  (2) 

- DUOS  (3) 

- I am not familiar with any of these tools  (4) 

| Page Break |  |
| --- | --- |

Q15 **Who will oversee DMS policy compliance at your institution?**

- Chief Information/Technology Officer  (1) 

- Institutional Signing Official(s)  (2) 

- Chief Compliance Officer  (3) 

- Other  (4) __________________________________________________

Q16 **Who will decide where data is deposited to meet DMS Policy requirements?**

- Individual investigators  (1) 

- Chief Compliance Officer  (2) 

- Institutional Signing Official  (3) 

- Chief Information/Technology Officer  (4) 

- Institutional Librarians  (5) 

- Other  (6) __________________________________________________

| Page Break |  |
| --- | --- |

Q17 **Would you be willing to pre-approve select researchers from your institution to request access to scientific data (not requiring review of each data access request before it goes to a data access committee)?**

- Yes  (1) 

- No  (2) 

Q18 **Would you be willing to pre-approve select researchers from your institution to deposit scientific data (not requiring review of each dataset submission to a repository)?**

- Yes  (1) 

- No  (2) 

End of Block: Block 1
